# Relationship between ultrasound elastography and myofibroblast distribution in breast cancer and its clinical significance

**DOI:** 10.1038/srep19584

**Published:** 2016-02-05

**Authors:** Yi Hao, Xia Guo, Binlin Ma, Lin Zhu, Lisha Liu

**Affiliations:** 1 Department of Ultrasound, the Affiliated Cancer Hospital of Xinjiang Medical University, Urumqi 830011, Xinjiang, China; 2 Central Laboratory of XinJiang Medical University/Collaborative Innovation Center of XinJiang Medical University, Urumqi 830011, Xinjiang, China; 3 Department of breast surgery, the Affiliated Cancer Hospital of Xinjiang Medical University, Urumqi 830011, Xinjiang, China; 4 Research Service Office, the Affiliated Cancer Hospital of Xinjiang Medical University, Urumqi 830011, Xinjiang, China

## Abstract

The study investigated the relationship between ultrasound elastography (USE) scoring and myofibroblast distribution with expression features of α-SMA + /CD34− in patients of Uyghur and Han ethnicities with breast masses in Xinjiang, China. The data was used to evaluate its clinical significance in the early diagnosis of breast cancer. A total of 300 patients with breast masses were included in the study, which involved conventional sonography and USE, with histopathologic diagnosis as the reference standard. Myofibroblast distribution was investigated by detecting the expression levels of α-SMA and CD34 in lesions using immunohistochemistry and real-time PCR. Out of 300 lesions, 185 were histologically malignant and 115 benign. The mean elasticity score for malignant lesions was significantly higher than for benign lesions. The expression level of α-SMA was elevated while the expression level of CD34 was lower in malignancies, compared with benign lesions. The expression of α-SMA was positively associated with the USE scores, while a negative relationship was observed between CD34 expression and USE scoring. The combination of USE and molecular diagnosis provides a promising modality for the early diagnosis and evaluation of the risks in particular types of breast cancer.

Ultrasound elastography (USE) is an imaging technique that can visualize tissue elasticity (stiffness) *in vivo* and provide additional information about breast lesions over conventional sonography and mammography. Many studies have reported that it can increase the specificity of conventional B-mode ultrasound (US) in the evaluation, characterization and differentiation of benign from malignant breast masses^1^,^2^,^3^,^4^. Two techniques are now available for clinical use: strain (compression-based elastography) and shear wave elastography. With either technique, acoustic information regarding lesion stiffness is converted into a black-and-white or color-scaled image that can also be superimposed on top of a B-mode gray-scale image.

Breast cancer is characterized by increasing stiffness of breast tissue; at physical examination, it has long been recognized that malignant tumors tend to feel hard compared with benign tumors. Breast elastography provides a non-invasive evaluation of the stiffness or hardness of breast mass, similar to a clinical palpation examination. Much data has been published that suggests that an association between breast tissue stiffness and breast cancer risk is biologically plausible. Norman *et al.* have suggested that the mechanical properties, especially stiffness, of the tissue might be associated with breast cancer risk^5^ and others have suggested that macroscopic stiffness of breast tumors predicts metastasis^6^.

The stromal microenvironment of tumor cells is different from that of normal cells. One of the features is the presence of many activated fibroblasts (termed myofibroblasts (MFS) or cancer-associated fibroblasts (CAFs)) in the tumor microenvironment (TME)^7^,^8^. Recent studies have shown that poorer prognosis in a wide range of tumor types is correlated with the presence of MFS in a neoplastic stroma^9^,^10^. MFS determines the fate of epithelial cells and promotes the malignant transformation of epithelial cells in a number of types of epithelial tumors, including breast, colorectal, prostate and lung cancer^11^,^12^. MFS contributes to the identification and diagnosis of benign and malignant disease. The stromal loss of CD34 expression (CD34−) and acquisition of smooth muscle actin (α-SMA + ) myofibroblastic features may constitute a prerequisite for tumor invasiveness in breast carcinoma^13^,^14^. MFS, namely, activated fibroblasts that express α-smooth muscle actin (α-SMA), produce collagen and extracellular matrix proteins and constitute the ‘desmoplastic reaction’ which may be important factors in altering the stiffness or elasticity of breast tissue^15^. Furthermore, the hyperplasia of MFS in breast cancer determines the stiffness of tumors. Therefore, MFSwith a signature of α-SMA + /CD34− is probably closely related to the indices of ultrasound elastography. The ability to synthesize breast US findings with multiple imaging modalities and clinical information is also necessary to ensure the best patient care.

The incidence of breast cancer is increasing year-by-year, seriously affecting the health of women in Xinjiang, a relatively geographically isolated region of China^16^,^17^. In many studies, there were no statistically significant differences in ages between the Uygur and Han patient populations^18^,^19^. However, patients in the Uygur population are often diagnosed with a larger tumor size, more metastatic axillary nodes, a longer time necessary for tumor excision and poor prognosis^20^,^21^, in comparison with the Han population. These findings indicate that the breast cancers in the Uyghur population may have specific characteristics, which should be considered in the treatment regimen. The aims of present study were to investigate the correlation between USE score and the expression of MFS in breast tumors, and to evaluate the clinical significance of USE and MFS in the early diagnosis and treatment of breast cancers, as well as comparing Uyghur and Han patients in Xinjiang.

## Patients and Methods

### Patients

A total of 300 patients with solid breast lesions, with a mean age of 44.8 ± 11.3 years (range 30 to 76 years), who were admitted to the Affiliated Cancer Hospital of Xinjiang Medical University, from May 2009 to Nov 2010, were included in this study. The basic characteristics of the patients are shown in Table 1. Prior written and informed consent was obtained from each participant, and the prospective study was approved by the Ethics Committee of the Affiliated Cancer Hospital of Xinjiang Medical University. The study was performed in accordance with relevant guidelines and regulations. Upon admission to the hospital, B-mode conventional sonography and USE examinations were performed, respectively. Examinations were performed prior to surgery, biopsy, or fine needle aspirations.

### Conventional sonography and USE

For each patient, bilateral whole-breast sonography in the transverse and longitudinal direction was carried out using a Hitachi EUB-8500 US scanner (Hitachi Medical, Tokyo, Japan) equipped with a 7.5–13.0 MHz linear-array transducer. The elasticity of the tissues was measured using the scanner equipped with a USE unit. B-mode conventional sonography and USE were performed at the same time by a single radiologist with 10 years experience in breast sonography. Pre-compression was not applied when obtaining the strain data^22^.

On conventional sonography, the lesion was localized, and then a region of interest (ROI) was placed surrounding the lesion, making sure the lesion area occupying no more than one third of the ROI. Lesion features, including shape, boundary, orientation, margin, echo pattern, posterior acoustic features and calcification, as well as surrounding tissue, were evaluated. The lesions were classified as category 2–5 lesions according to the 2nd edition of the Breast Imaging Recording and Data System (BI-RADS) criteria^23^. Category 1 and 2 are benign, category 3 is “probably benign” (negative), category 4 is “suspicious” and category 5 is “highly suggestive of malignancy”.

The USE images were obtained together with B-mode images in the real-time screen and included the lesion and surrounding tissue. These images were color coded and translucently superimposed on the B-mode images. USE images were obtained by moving the probe around in the ROI, with appropriate compression (the indicator bar displaying 3 or 4). These images are shown from red to blue; the softest component is shown in red, with the greatest strain, whereas the hardest component with no strain is exhibited in blue, and green indicated intermediate elasticity.

**Figure 1:**
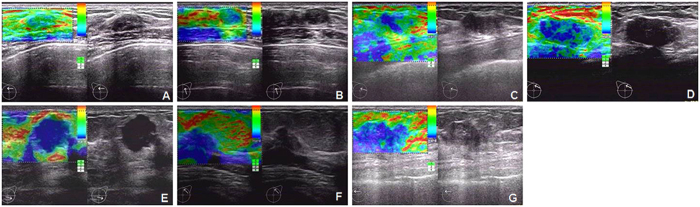
Representative pictures for the USE diagnosis (7-point elasticity scoring) of benign and malignant breast lesions. Scores <5 were classified as benign lesions, whereas scores ≥5 were classified as malignant lesions. (**A**) Elastosonography shows uniformed green (Score 1). Pathologic result: benign. (**B**) Elastosonography shows blue and green mosaic. Main color is green (Score 2). Pathologic result: benign. (**C**) Elastosonography shows center blue with green surrounding (Score 3). Pathologic result: benign. (**D**) Elastosonography shows blue and green mosaic. Main color is blue (Score 4). Pathologic result: benign. (**E**) Elastosonography shows lump was covered in blue area. (Score 5). Pathologic result: infiltrating ductal carcinoma. (**F**) Elastosonography shows lump its below was covered in blue area (Score 6). Pathologic result: infiltrating ductal carcinoma. (**G**) Elastosonography shows lump its surrounding was covered in blue area. Blue area is bigger than lump area (Score 7). Pathologic result: infiltrating ductal carcinoma.

The qualitative evaluation of the USE images was achieved on the basis of a 7-point scoring system^1^. A score of 1 indicated a lesion evenly shaded in green; 2 indicated a lesion with a mosaic pattern of green and blue (mostly green); 3 indicated a lesion with blue central part and green periphery; 4 indicated a lesion with a mosaic pattern (mostly blue); 5 indicated a lesion (excluding the peripheral area) with blue; 6 indicated an entire lesion with blue central part and periphery; 7 indicated a lesion with blue in periphery and most of the inside part, with a small blue part inside the lesion (Fig. 1). Scores <5 were classified as benign lesions, whereas scores ≥5 were classified as malignancy.

### E/B ration analysis

The longitudinal dimension of the lesion was measured on the B-mode and the longitudinal elastogram dimension (E) was compared to the B-mode dimension (B) to form an E/B ratio. According to the literature, E/B ratios >1 correspond to malignant lesions^24^.

### Data analysis

Interpretation of US examinations was compared with the histological findings, with regards to the sensitivity, specificity, positive and negative predictive values (PPV and NPV), and false-positive and false-negative rates. The following equations were used for the evaluation: sensitivity = patients with suspected breast cancer/patients with histologically confirmed breast cancer; specificity = patients with suspected benign disease/patients with histologically confirmed benign disease; positive predictive value (PPV) = patients with histologically confirmed breast cancer/patients with suspected breast cancer; negative predictive value (NPV) = patients with histologically confirmed benign disease/patients with suspected benign disease. A false-negative result indicated that the US examination classified a benign lesion, which was histologically confirmed as a malignancy. A false-positive result indicated that US examination identified a malignancy, which was histologically confirmed as a benign lesion. We obtained the false-positive and false-negative rates and compared the performances of conventional sonography and USE in diagnosing benign and malignant lesions.

### Histopathologic diagnoses

The final diagnosis was determined by histopathology after surgical excision or US-guided core needle biopsy (BARD MAGNUM Reusable Core Biopsy Instrument with MN1620 (16 gauge) biopsy needles (Bard Peripheral Vascular, Inc., Tempe, AZ, USA). Histopathologic diagnoses of the specimens were obtained and served as reference standards. All diagnoses were made by a specialized breast pathologist with 25 years experience, who was blinded to the results of US.

### Core biopsies

One of five radiologists specializing in breast imaging performed all of the biopsies. Prior to biopsy, a breast ultrasonography (including the bilateral axillae) was performed and also a Color Doppler Ultrasound Diagnostic System (HITACHI EUB—8500)-guided core biopsy. A minimum of 5 biopsy samples was obtained, with additional samples collected at the discretion of the radiologist. The pathologic results for each case were retrospectively reviewed with the final pathology findings as determined after breast surgery. Immunohistochemistry was used to identify intact myoepithelial cells after CD34 and α-SMA staining.

### Immunohistochemistry

Paraffin-embedded tissues were cut into 4-μm sections and baked at 65 °C for 30 min. After being deparaffinized and rehydrated, the sections were submerged in EDTA (pH 8.0) and autoclaved for antigen retrieval, and then treated with 3% H_2_O_2_, followed by incubation with 1% FBS. Mouse anti-human anti-α-SMA monoclonal primary antibody (1:50 dilution; Santa Cruz, Santa Cruz, CA, USA) or anti-CD34 polyclonal antibody (1:100 dilution; Santa Cruz) was added for incubation at 4 °C overnight. Horseradish peroxidase (HRP)-labeled secondary antibody (ZSGB-BIO, Beijing, China) was applied and tissues incubated at room temperature for 30 min, followed by 5 min incubation with DAB at room temperature for color development. Then the sections were counterstained with hematoxylin and mounted using Permount Medium (BIOS, Beijing, China). The sections were visualized and photographed under a light microscope. The proportion of positively stained cells was graded as follows: 0 (≤5% positively stained cells), 1(>5–25% positively stained cells), 2 (>25–75% positively stained cells), and 3 (>75% positively stained cells). The staining intensity was determined on a scale of 0 (no staining), 1 (weak staining, light yellow), 2 (moderate staining, yellowish brown) and 3 (strong staining, brown). The staining index was calculated according the follow equation: staining index = (staining intensity × proportion of positively stained cells)/2. The sum of both scores was used to identify the expression grades: 0–1 indicated negative expression; 2–4 indicated weak expression; 5–8 indicated moderate expression; ≥9 indicated high-level expression.

### Real-time PCR

Total RNA was extracted using the Triazol reagent (Invitrogen, Carlsbad, CA, USA). After spectrophotometric quantification, 1 μg total RNA was used for reverse transcription in a final volume of 20 μL with AMV reverse transcriptase (Promega, Madison, WI, USA), according to the manufacturer’s instructions. Real-time PCR was performed using the iQ5 96 real-time quantitative PCR detection system (BioRad, Hercules, CA, USA). 25 μL reaction system contained the corresponding cDNA, forward and reverse primers, and SYBR-Green PCR Master Mix (Promega). Primer sequences were α-SMA FW 5′-CGAGAGGACGTTGTTAGCATAGAG-3′, α-SMA RV 5′-GGGCATCCACGAAACCA-3′; CD34 FW 5′-ATTGAGAAACG ATTTG CCTACA-3′, CD34 RV 5′ GGGAAATGTTCTCCTTTGCTT-3′; β-actin FW 5′-AAGGACCTGTATGCCAACACA-3′, β-actin reverse 5′-ATCCACACAGAATA CTTGCGTT-3′ and β-actin was used as the control. Relative expression levels of the target genes were determined using the ∆∆Ct method (iQ5 optical system software, version 2.1.)

### Statistical analysis

Statistical analyses were performed with SPSS version 17 for Windows (SPSS Inc., Chicago, IL, USA). Differences among elasticity scores for benign and malignant breast lesions were assessed by the Student’s *t*-test. The McNemar test was performed to determine the sensitivity, specificity, positive and negative predictive values of the ultrasound examination. Relationships between the stiffness values and the histological features were compared using the independent *t*-test or one-way ANOVA. Clinical and histological variables were compared among the tumor subtypes using the χ^2^ test or Fisher’s exact test. *P* < 0.05 was considered to be statistically significant.

## Results

In our study, we included a total of 300 patients with breast masses, from which 198 were of Han and 102 of Uygur ethnicity; 185 cases were diagnosis with breast cancer (63 Uyghur and 122 Han patients), while the other 115 cases were diagnosed as benign lesions (39 Uyghur and 76 Han patients), based on the pathological diagnosis. The mean age of patients with malignant lesions and the frequency of malignancy compared to benign lesions were significantly higher (0 < 0.05) in both ethnic groups. Grade I and grade III, as well as tumor sizes <10 mm and >30 mm, and the ratio of palpable tumors, differed significantly between the Han and Uyghur patients (Table 1).

### Histological diagnosis of patients with breast lesions

The final histological diagnosis of patients with breast lesions is shown in Table 2. The results from histologic diagnosis after complete excision served as the reference standards. In the following sections, diagnoses from conventional US and USE (elasticity scoring) were analyzed and compared with the histologic diagnosis.

### USE diagnosis (7-point elasticity scoring) of Uyghur and Han patients with breast lesions

**Figure 2:**
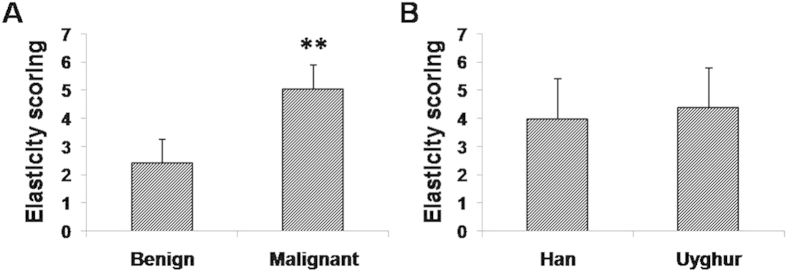
USE diagnosis (7-point elasticity scoring) of Uyghur and Han patients with breast lesions. (**A**) Elasticity scores for benign and malignant lesions in these patients. Compared with the benign lesions, ^*^P < 0.05. (**B**) Distribution of elasticity scores of the Uyghur and Han population.

The distribution of elasticity scores for benign and malignant lesions in patients are shown in Fig. 2 and Table 3 (in comparison with the histological diagnosis), respectively. The mean elasticity score for malignant lesions (5.05 ± 0.85) was significantly higher than that for benign lesions (2.44 ± 0.82) (Fig. 2A, *P* < 0.01). Moreover, the mean elasticity score of the Uyghur population appeared to be slightly higher than the Han population, but statistical significance was not reached (Fig. 2B, *P* > 0.05). As shown in Table 3, the sensitivity, specificity, positive and negative predictive values, and false-positive and false-negative rates for USE diagnosis were 88.6%, 86.1%, 91.1%, 82.5%, 13.9% and 11.4%,respectively. When the cutoff point between scores 4 and 5 was used, the 7-point elasticity scoring system yielded 16 false-positive cases (including 2 cases of inflammatory lesions, 9 cases of fibroadenoma, 1 case of focal adenosis, and 4 cases of intraductal papilloma), as well as 21 false-negative results (including 2 cases of ductal carcinoma *in situ* (DCIS) 12 cases of invasive ductal carcinoma, 1 case of invasive lobular carcinoma, 3 cases of mixed carcinomas and 3 cases of special type carcinoma).

The elasticity scoring of various malignant lesions confirmed by histopathological diagnosis are shown in Table 4. In total, 21 of the 185 malignant lesion were misdiagnosed as benign by the elasticity score determination.

**Figure 3:**
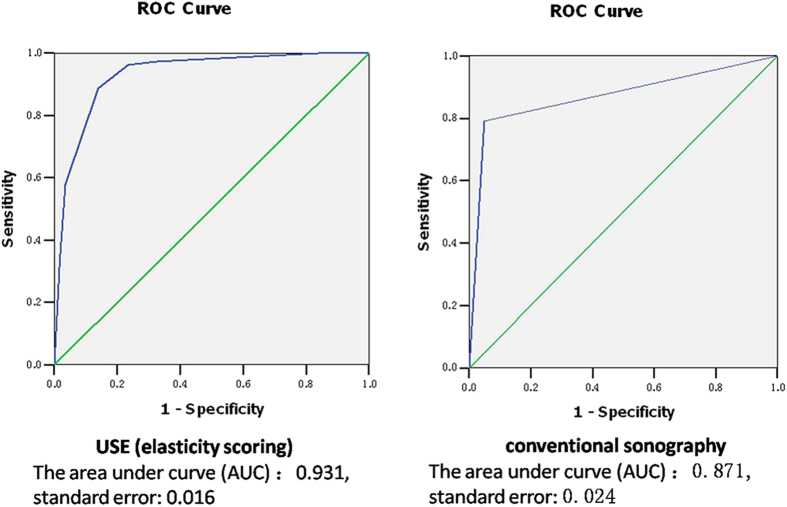
Receiver operating characteristic (ROC) curves for the conventional sonography and USE in diagnosing benign and malignant breast lesions.

To evaluate the performance of the USE diagnosis of patients with breast disease, conventional sonography was used for the comparison. Figure 3 shows the receiver operating characteristic (ROC) curves for conventional sonography and USE, in differentiating breast cancers from benign lesions. The area under the curve (AUC) for USE (0.931) was significantly higher than for conventional sonography (0.871, *P* < 0.05). In addition, the comparisons of the sensitivity, specificity, and positive and negative predictive values of the conventional sonography and USE in diagnosing benign and malignant lesions in Uyghur and Han patients, respectively, are shown in Table 5. USE was superior to conventional sonography in diagnosing benign and malignant lesions in all respects in both the Uyghur and Han populations. Taken together, these results indicate that USE can achieve high accuracy in diagnosing benign and malignant breast lesions, which was significantly superior to the conventional sonography BI-RADS classification in the diagnosis of breast lesions.

### Expression levels of α-SMA and CD34 in Uyghur and Han patients with breast lesions

**Figure 4:**
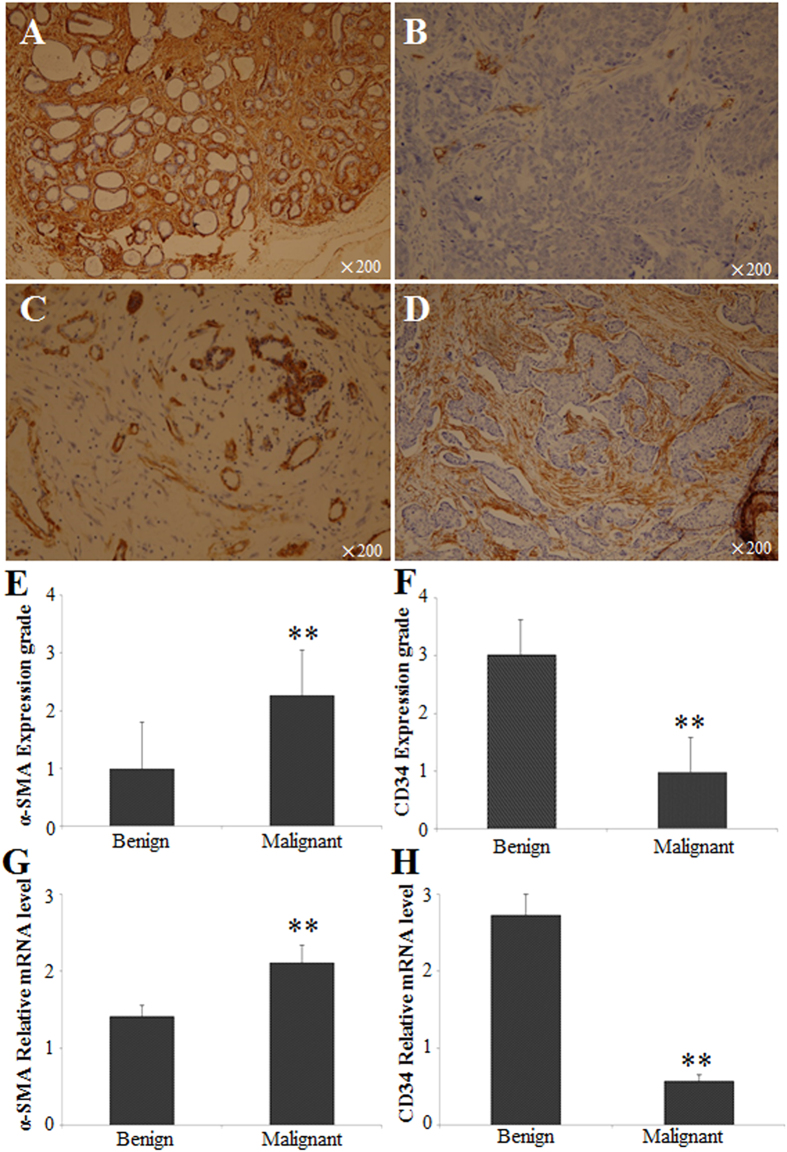
Expressions of α-SMA and CD34 in patients with benign and malignant breast lesions (**A**) The expression of CD34 in benign breast lesions stromal cells shows strong positive signal. (**B**) The expression of CD34 in breast cancer stromal cells shows negative signal. It is expressed in blood vessel. (**C**) The expression of α-SMA in benign breast lesions stromal cells shows negative signal. It is expressed in ductal myoepithelial cells. (**D**) The expression of α-SMA in breast cancer stromal cells shows strong positive signal. Magnification: 200×, staining method: PV9000. Compared with the benign lesions, ^**^P < 0.01.

**Figure 5:**
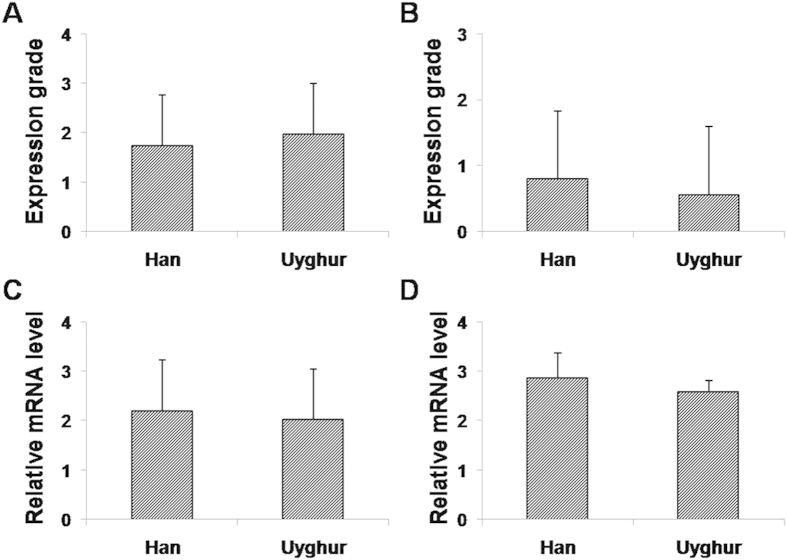
Expressions of α-SMA and CD34 in the Uyghur and Han patients with breast lesions. The expression levels of α-SMA and CD34 were detected by immunohistochemistry (**A,B**) and real-time PCR (**C,D**), respectively, in Uyghur and Han patients with breast lesions. (**A,B**) The protein expression levels of α-SMA (**A**) and CD34 (**B**) were detected by immunohistochemistry in Uyghur and Han patients. (**C,D**)The mRNA expression levels of α-SMA (**C**) and CD34 (**D**) were detected by real-time PCR in Uyghur and Han patients. Compared with the benign lesions, ^*^P < 0.05.

To investigate MFS distribution in breast lesions in Uyghur and Han patients, the protein and mRNA expression levels of α-SMA and CD34 were measured using immunohistochemistry and real-time PCR. The results showed that in benign breast tissues, α-SMA was mainly expressed in ductal myoepithelial cells (brown granules), rather than in stroma; however, in breast cancer tissues, positive staining of α-SMA was observed in stroma, without expression in ductal myoepithelial cells (Fig. 4A,B). Moreover, CD34 was mainly located in stroma in benign breast tissues, while in malignant lesions, CD34 was mainly found in blood vessels, with little expression in the stroma (Fig. 4C,D). In addition, as shown in Fig. 4E,F, the expression rate of α-SMA in the benign lesions was significantly lower than that in malignancy (*P* < 0.01), while the expression grade of CD34 in benign breast tissues was dramatically higher than in breast cancers (*P* < 0.01). Our results from real-time PCR showed that the mRNA expression level of α-SMA was significantly elevated while the CD34 mRNA expression level was significantly reduced in malignancies, compared with benign lesions (Fig. 4G,H, both *P* < 0.01). No significant differences in the protein and mRNA expression levels of α-SMA or CD34 were observed between the Uyghur and Han populations (Fig. 5, *P* > 0.05). Taken together, these results suggest that significant differences exist in the expression levels of α-SMA and CD34 in benign and malignant lesions.

### Correlation analysis of USE and α-SMA/CD34 expression in breast lesions

**Figure 6:**
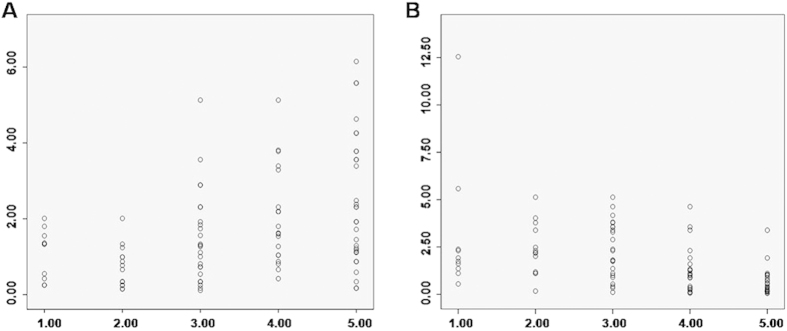
Correlation analysis of elasticity scores and α-SMA (**A**) and CD34 (**B**) expressions in breast lesions.

Next, the relationship between USE and the expression levels of α-SMA and CD34 in breast lesions was investigated. As described above, the elasticity score for malignant lesions was significantly higher than for benign lesions. In addition, the expression level of α-SMA was elevated, while the expression level of CD34 was decreased in malignancies, compared with benign lesions (Table 6). Correlation analysis showed that the protein and mRNA expression levels of α-SMA in the breast masses were positively associated with the USE scores (*P* < 0.01, *rs* = 0.406). Moreover, a negative relationship was observed between the protein and mRNA expression levels of CD34 and USE scoring (*P* < 0.01, *rs* = −0.596) (Fig. 6 and Table 7).

## Discussion

From 1991, USE has been proposed and introduced as a reliable method to evaluate tissue stiffness^25^. Krouskpo *et al.*^26^ reported different elasticity coefficients for various breast tissues, in descending order: invasive ductal carcinoma, non-invasive ductal carcinoma, breast fibrosis, breast, and adipose tissues. Many studies found that higher lesion stiffness values correlated with high histological grades of breast cancer, which may improve the prediction of breast cancer risk in individuals^5^. A possible reason for the high correlation in the malignant group was that high stiffness malignant lesions might have a more obvious desmoplastic reaction or cancerous infiltration in the peritumoral region^27^,^28^. Based on the theory of elasticity, USE provides an alternative option for the identification of benign and malignant breast lesions^29^,^30^. In the present study, the USE-based 7-point elasticity scoring system and the conventional sonography BI-RADS grading system were used to classify solid breast lesions in the Uyghur and Han population in Xinjiang, Northwest China. Our results indicated that USE was superior to conventional sonography in identifying benign and malignant breast lesions and had a significantly higher accuracy. However, the stability of USE has yet to be proven, and due to the difficulty in manipulation, a combination of USE with conventional sonography would achieve a more satisfactory accuracy in the diagnosis of benign and malignant breast lesions.

Elasticity scoring of various malignant lesions confirmed by histopathological diagnosis showed that invasive carcinomas were associated with increased stiffness: out of 130 cases of invasive ductal carcinoma, 118 cases were scored ≥5 (118/130, 90.1%); 9 cases was diagnosed as invasive lobular carcinoma, in which 8 cases were scored ≥5 (8/9,88.9%); in 25 cases of mixed carcinomas (mainly invasive ductal carcinoma), 22 cases had a score of ≥5 (22/25, 88.0%); in the other 11 special types of lesions, 8 cases were scored ≥5 (8/11, 72.7%) and of 10 DCIS cases 8 were scored ≥5 (8/10, 80.0%). These results suggest that invasive carcinomas and several special types of cancers exhibited higher harnesses’, which could be more easily identified by USE, which is in agreement with previous studies^5^,^31^. The total sensitivity of our USE scoring was 88.6% for malignant and 86.1% for benign lesions. In order to compare our USE results with E/B ratios, we found that from 185 malignant lesions, 168 had an average E/B ratio of 1.380 ± 0.303, resulting in a sensitivity of 90.8%, which is similar to the USE results.

To date, an abundance of data have supported the crucial roles of TME in providing cancer cells with proliferative, migratory, survival and invasive propensities, favoring the processes of tumorigenesis^7^. The cancerous reactive stroma is frequently populated by a large number of MFS. In malignant tissues, MFS were mainly distributed in the tumor invasion front and tumor-stroma interface, or near the vascular endothelial cells in stroma, surrounding the cancer nest. Recent studies have shown that MFS could also exert promoting effects in breast cancers, whose biological characteristics are particularly different from normal breast fibroblasts. The stromal loss of CD34 expression and the acquisition of α-SMA myofibroblastic features may constitute a prerequisite for tumor invasiveness in breast carcinoma^14^. Our results show that the expression of α-SMA was positively associated with USE scoring in breast lesions, indicating that USE might reflect the distribution of MFS in breast masses to a certain degree. The stiffness of tumors may well be decided by the aggregated distribution of cancer-stromal MFS. However, it is difficult to distinguish breast cancers from sclerosing adenosis with USE and α-SMA is also expressed in sclerosing adenosis, which might interfere with the consistency between USE scoring and myofibroblast distribution. Therefore, the detection of CD34 expression was introduced into our study. CD34 is a transmembrane glycoprotein, primarily located in hematopoietic precursor cells, endothelial cells, and undifferentiated mesenchymal cells in various tissues, including the breasts^32^. It has been found that changes in the stroma could activate the naive mesenchymal cells, with decreased CD34 expression and elevated α-SMA expression, which finally differentiated into myofibroblasts^33^,^34^. On the other hand, there are a large number of fibroblasts expressing CD34 in sclerosing adenosis.

Our results have shown that both protein and mRNA expression levels of α-SMA were significantly upregulated in malignancies, but the protein and mRNA expression levels of CD34 were significantly higher in benign compared to malignant lesions. Moreover, the association analysis showed that the expression of α-SMA was positively associated with the USE scores, while a negative relationship was observed between CD34 expression and USE scoring. Taken together, these results suggest that the distribution of MFS in breast masses may well contribute to increased stiffness.

According to the USE scoring of breast masses, no significant differences were observed in malignancy between the Uyghur and Han populations. However, our results have shown that the elasticity score of the Uyghur population appeared to be slightly higher than the Han population (even though statistical significance was not reached), indicating that the Uyghur population had breast lesions with higher stiffness. This phenomenon might be due to that fact that Uyghur women usually have larger volume breasts, with a thick layer of fat and a thin layer of glands, while Han women often have relatively small volume breasts, with less adipose tissue and more glands. Therefore, the elastic ratio of breast lesions to normal tissues in the Uyghur women might be higher than in Han women.

In conclusion, USE scores can diagnose malignant lesions with a high sensitivity. Higher USE scores in malignant tumors might reflect a higher portion of MFS with acquisition of smooth muscle actin and a loss of CD34 expression. The combination of USE and molecular diagnosis might provide a promising modality in the early diagnosis and evaluation of the risk of breast cancers.

## Additional Information

**How to cite this article**: Hao, Y. *et al.* Relationship between ultrasound elastography and myofibroblast distribution in breast cancer and its clinical significance. *Sci. Rep.* **6**, 19584; doi: 10.1038/srep19584 (2016).

## Figures and Tables

**Table 1:** Clinical information on 300 patients.

Variables	Uyghur patients (*n* = 102)		Han patients (*n* = 198)	
	Benign lesions*n* = 39	Malignant lesions*n* = 63	Benign lesions*n* = 76	Malignant lesions*n* = 122
Mean Age	38.7 ± 11.1	49.8 ± 9.8*	37.2 ± 10.6	53.2 ± 10.2*
Histologic grade				
I grade		6 (9.52%)		28 (22.95%)#
II grade		38 (60.32%)		70 (57.38%)
III grade		19 (30.16%)		24 (19.67%)#
Tumor size (mm)				
<10	1 (0.98%)	4 (3.92%)	10 (5.05%)#	8 (4.04%)#
10–20	16 (15.69%)	22 (21.57%)	31 (15.67%)	65 (32.83%)
20–30	13 (12.75%)	23 (22.54%)	26 (13.13%)	40 (20.20%)
>30	9 (8.82%)	14 (13.73%)	9 (4.54%)#	9 (4.54%)#
Triple negative tumors		11 (17.46%)		13 (10.65%)#
Palpable	33 (32.35%)	58 (56.86%)	37 (18.69%)*#	86 (43.43%)*#
Frequency	39 (38.24%)	63 (61.76%)*	76 (38.39%)	122 (61.61%)*

Compared to benign group,*P < 0.05.

Compared to Uyghur group, ^#^*P* < 0.05.

**Table 2:** Histological diagnosis of malignant and benign lesions in 300 breast lesions.

Histological diagnosis	N
Malignant lesions	185
Ductal carcinoma *in situ* (DCIS)	10
Invasive ductal carcinoma	130
Invasive lobular carcinoma	9
Mixed carcinomas	25
Special types	11
Benign lesions	115

**Table 3:** Sonoelastographic diagnosis of malignant and benign lesions in 300 breast lesions.

Histologicaldiagnosis	Sonoelastographic diagnosis			Sensitivity(%)
	Malignantlesions	Benignlesions		
Malignant lesions	185	164	21	88.6
Benign lesions	115	16	99	86.1

**Table 4:** Elasticity scoring of malignant lesions confirmed by histological diagnosis.

Malignant lesions	Han	Elasticity Score								Elasticity Score						
		1	2	3	4	5	6	7	Uyghur	1	2	3	4	5	6	7
Ductal carcinoma *in situ* (DCIS)	8				**1**	5	2		2				**1**	1		
	6.56%				0.82%	4.10%	1.64%		3.18%				1.59%	1.59%		
Invasive ductal carcinoma	83			**3**	**4**	27	31	18	47			**2**	**3**	11	20	11
	68.03%			2.46%	3.28%	22.13%	25.41%	14.75%	74.60%			3.17%	4.76%	17.46%	31.75%	17.46%
Invasive lobular carcinoma	6					2	3	1	3				**1**	1		1
	4.92%					1.64%	2.46%	0.82%	4.77%				1.59%	1.59%		1.59%
Mixed carcinomas	18				**3**	3	5	7	7					2	3	2
	14.76%				2.46%	2.46%	4.10%	5.74%	11.11%					3.17%	4.76%	3.17%
Special types	7		**1**		**1**		3	2	4			**1**		1	2	
	5.74%		0.82%		0.82%		2.46%	1.64%	6.35%			1.59%		1.59%	3.17%	

The cases marked bold were malignant lesions with elasticity scores <5 (per definition cases with elasticity scores ≥5 were considered as malignant lesions and cases with elasticity scores <5 as benign lesions).

**Table 5:** Sensitivity, specificity and positive and negative predictive values of USE and conventional US in diagnosing benign and malignant lesions in Uyghur and Han patients.

Population	Diagnosis	Sensitivity (%)	Specificity (%)	PPV (%)	NPV (%)
Uyghur	Conventional US	84.4	71.9	84.6	78.1
	USE	90.7	85.9	92.9	81.0
Han	Conventional US	81.2	75.1	80.1	77.3
	USE	88.0	86.3	90.6	82.8

**Table 6:** The expression levels of CD34 and α-SMA in the benign and malignant groups.

Group	CD34				a-SMA			
	0	1	2	3	0	1	2	3
Benign	12	17	48	14	32	34	19	6
Malignant	160	18	24	7	16	28	81	84
*Z*	−10.258				−8.478			
*P*	0.000				0.000			

**Table 7:** Stiffness correlation with gain of α-SMA and loss of CD34.

Correlative factor	Elasticity imaging score (*γ*_*s*_)	*P value*
CD34	−0.62	0.000
α-SMA	0.487	0.000

Table CD34, α-SMA expression level and Elasticity imaging score.

## References

[b1] Itoh A. *et al.* Breast disease: clinical application of US elastography for diagnosis. Radiology. 239, 341–50 (2006). 16484352 10.1148/radiol.2391041676

[b2] Cho N. *et al.* Nonpalpable breast masses: evaluation by US elastography. Korean J Radiol. 9, 111–8(2008). 18385557 10.3348/kjr.2008.9.2.111 PMC2627231

[b3] Cho N. *et al.* Distinguishing benign from malignant masses at breast US: combined US elastography and color doppler US–influence on radiologist accuracy. Radiology. 262, 80–90(2012). 22084209 10.1148/radiol.11110886

[b4] Yi A. *et al.* Sonoelastography for 1,786 non-palpable breast masses: diagnostic value in the decision to biopsy. Eur Radiol. 22, 1033–40 (2012). 22116557 10.1007/s00330-011-2341-x

[b5] Boyd N. F. *et al.* Evidence that breast tissue stiffness is associated with risk of breast cancer. PloS one. 9, e100937 (2014). 25010427 10.1371/journal.pone.0100937 PMC4091939

[b6] Fenner J. *et al.* Macroscopic stiffness of breast tumors predicts metastasis. Sci Rep. 4, 5512 (2014). 24981707 10.1038/srep05512 PMC4076689

[b7] Polanska U. M. & Orimo A. Carcinoma-associated fibroblasts: non-neoplastic tumour-promoting mesenchymal cells. J Cell Physiol. 228, 1651–7 (2013). 23460038 10.1002/jcp.24347

[b8] Polanska U. M., Acar A. & Orimo A. Experimental generation of carcinoma-associated fibroblasts (CAFs) from human mammary fibroblasts. J Vis Exp. JoVE. 56, e3201 (2011). 22064505 10.3791/3201 PMC3227206

[b9] Brentnall T. A. *et al.* Arousal of cancer-associated stroma: overexpression of palladin activates fibroblasts to promote tumor invasion. PloS one. 7, e30219 (2012). 22291919 10.1371/journal.pone.0030219 PMC3264580

[b10] Goicoechea S. M. *et al.* Palladin promotes invasion of pancreatic cancer cells by enhancing invadopodia formation in cancer-associated fibroblasts. Oncogene. 33, 1265–73 (2014). 23524582 10.1038/onc.2013.68 PMC3912215

[b11] Mehner C. & Radisky D. C. Triggering the landslide: The tumor-promotional effects of myofibroblasts. Exp Cell Res. 319, 1657–62 (2013). 23528452 10.1016/j.yexcr.2013.03.015 PMC3748147

[b12] Otranto M. *et al.* The role of the myofibroblast in tumor stroma remodeling. Cell Adh Migr. 6, 203–19 (2012). 22568985 10.4161/cam.20377 PMC3427235

[b13] Catteau X., Simon P., Vanhaeverbeek M. & Noel J. C. Variable stromal periductular expression of CD34 and smooth muscle actin (SMA) in intraductal carcinoma of the breast. PloS one. 8, e57773 (2013). 23469238 10.1371/journal.pone.0057773 PMC3585862

[b14] Catteau X., Simon P. & Noel J. C. Myofibroblastic stromal reaction and lymph node status in invasive breast carcinoma: possible role of the TGF-β1/TGF-βR1 pathway. BMC cancer. 14, 499 (2014). 25011545 10.1186/1471-2407-14-499 PMC4099161

[b15] Satish L. *et al.* Reversal of TGF-beta1 stimulation of alpha-smooth muscle actin and extracellular matrix components by cyclic AMP in Dupuytren’s-derived fibroblasts. BMC Musculoskelet Disord. 12, 113 (2011). 21612641 10.1186/1471-2474-12-113 PMC3125251

[b16] Cheng F., Zhang Q., Liu W. & Li D. Ciinicopathologic Analysis of 2019 Breast Cancer Patients with Han and Uygur Nationalities in Xi jiang Area. Cancer Res ON Prevention And Treatment. 37, 1312–14 (2010).

[b17] Li S. *et al.* Analysis of clinical characteristics and related factors of breast cancer patients with different nationalities in Xinjiang areas. J Xinjiang Med Univ. 35, 895–901 (2012).

[b18] Yi H., Xiaoyu L. & Lisha L. Study on the correlation and clinical significancebetween ultrasound elastography and thedistribution of myofibroblast in breasttumor. J Xinjiang Med Univ. 21, 51–54 (2012).

[b19] Yi H. & Li G. Ultrasonic evaluation the value of the neoadjuvant Chemotherapy’s curative effect to breast cancer. J Xinjiang Med Univ. 32, 768–770 (2014).

[b20] Yi H. & Li G. General Ultrasonography,elastosonography and their correlation in differential diagnosis of breast masses. J Xinjiang Med Univ. 37, 1326–1328 (2014).

[b21] Wang C. H., Li J. Z. & Zhang W. Breast cancer molecular subtypes of Uygur and Han in Xinjiang of China. Int J Clin Exp Med. 7, 1116–21 (2014). 24955192 PMC4057871

[b22] Barr R. G. & Zhang Z. Effects of precompression on elasticity imaging of the breast: development of a clinically useful semiquantitative method of precompression assessment. J Ultrasound Med. 31, 895–902 (2012). 22644686 10.7863/jum.2012.31.6.895

[b23] Mendelson E. *et al.* ACR BI-RADS^®^ Ultrasound. ACR BI-RADS^®^ Atlas, Breast Imaging Reporting and Data System. (Reston, VA, American College of Radiology, 2013).

[b24] Barr R. G., Grajo J. R. & Youngstown O. H. Predictive value of EI/B-mode ratio in strain elastography to predict breast cancer tumour grade. EPOS Eur Society of Radiol. 23, C-1823 (2012).

[b25] Ophir J. *et al.* Elastography: a quantitative method for imaging the elasticity of biological tissues. Ultrasonic imaging. 13, 111–34 (1991). 1858217 10.1177/016173469101300201

[b26] Krouskop T. A. *et al.* Elastic moduli of breast and prostate tissues under compression. Ultrasonic imaging. 20, 260–74 (1998). 10197347 10.1177/016173469802000403

[b27] Schaefer F. K. *et al.* Breast ultrasound elastography--results of 193 breast lesions in a prospective study with histopathologic correlation. Eur J Radiol. 77, 450–6 (2011). 19773141 10.1016/j.ejrad.2009.08.026

[b28] Youk J. H. *et al.* Shear-wave elastography of invasive breast cancer: correlation between quantitative mean elasticity value and immunohistochemical profile. Breast Cancer Res Treat. 138, 119–26 (2013). 23324903 10.1007/s10549-013-2407-3

[b29] Zhao Q. L. *et al.* Diagnosis of solid breast lesions by elastography 5-point score and strain ratio method. Eur J Radiol. 81, 3245–9 (2012). 22749109 10.1016/j.ejrad.2012.06.004

[b30] Stachs A. *et al.* Differentiating between malignant and benign breast masses: factors limiting sonoelastographic strain ratio.Ultraschall Med. 34, 131–6 (2013). 23108926 10.1055/s-0032-1313168

[b31] Chang J. M. *et al.* Stiffness of tumours measured by shear-wave elastography correlated with subtypes of breast cancer. Eur Radiol. 23, 2450–8 (2013). 23673574 10.1007/s00330-013-2866-2

[b32] Orecchioni S. *et al.* Complementary populations of human adipose CD34+ progenitor cells promote growth, angiogenesis, and metastasis of breast cancer. Cancer Res. 73, 5880–91 (2013). 23918796 10.1158/0008-5472.CAN-13-0821

[b33] Ho S. K. *et al.* Phyllodes tumours of the breast: the role of CD34, vascular endothelial growth factor and β-catenin in histological grading and clinical outcome. Histopathology. 63, 393–406 (2013). 23772632 10.1111/his.12177

[b34] Catteau X., Simon P. & Noel J. C. Myofibroblastic reaction is a common event in metastatic disease of breast carcinoma: a descriptive study. Diagn Pathol. 9, 196 (2014). 25339428 10.1186/s13000-014-0196-6 PMC4220068

